# Monitoring SF_6_ Gas Leakage Based on a Customized Binocular System

**DOI:** 10.3390/s24030993

**Published:** 2024-02-03

**Authors:** Wenrong Si, Yingying Zhao, Yan Wang, Ben Li, Geng Tong, Yiting Yu

**Affiliations:** 1 State Grid Shanghai Electric Power Research Institute, Shanghai 200437, China; siwenrong@126.com (W.S.); zhaoyy_sh@163.com (Y.Z.); 2 Ningbo Institute of Northwestern Polytechnical University, School of Mechanical Engineering, Northwestern Polytechnical University, Xi’an 710072, China; wy26@mail.nwpu.edu.cn (Y.W.); liben0709@mail.nwpu.edu.cn (B.L.); tonggeng@mail.nwpu.edu.cn (G.T.); 3 Key Laboratory of Micro/Nano Systems for Aerospace (Ministry of Education), Key Laboratory of Micro- and Nano-Electro-Mechanical Systems of Shaanxi Province, Northwestern Polytechnical University, Xi’an 710072, China

**Keywords:** infrared imaging, customized, binocular system, SF_6_ gas detection

## Abstract

Sulfur hexafluoride (SF_6_) gas is extensively utilized as an insulating and arc-quenching medium in the circuit breakers and isolating switches of electrical equipment. It effectively isolates the circuits from the atmosphere and promptly extinguishes arcs. Therefore, the issue of SF_6_ gas leakage poses a significant threat to the related application fields, and the detection of SF_6_ gas leakage becomes extremely important. Infrared imaging detection offers advantages including non-contact, high precision, and visualization. However, most existing infrared detection systems are equipped with only one filter to detect SF_6_ gas. The images captured contain background noise and system noise, making these systems vulnerable to interference from such noises. To address these issues, we propose a method for monitoring SF_6_ gas leakage based on a customized binocular imaging (CBI) system. The CBI system has two filters, greatly reducing the interference of system noise and background noise. The first filter features the absorption resonant peak of SF_6_ gas. The second filter is used to record background noise and system noise. One aspect to note is that, in order to avoid the interference of other gases, the central wavelength of this second filter should keep away from the absorption resonant peaks of those gases. Accordingly, the central wavelengths of our customized filters were determined as 10,630 nm and 8370 nm, respectively. Then, two cameras of the same type were separately assembled with a customized filter, and the CBI prototype was accomplished. Finally, we utilized the difference method using two infrared images captured by the CBI system, to monitor the SF_6_ gas leakage. The results demonstrate that our developed system achieves a high accuracy of over 99.8% in detecting SF_6_ gas. Furthermore, the CBI system supports a plug-and-play customization to detect various gases for different scenarios.

## 1. Introduction

Sulfur hexafluoride (SF_6_) gas is widely used as an insulating and arc-quenching medium in the power industry, particularly in high-voltage electrical equipment, due to its excellent properties [1,2,3]. Thus, with the rapid development of electrical systems, the issue of gas leakage has become increasingly significant for SF_6_ in various application areas. Such gas leakage not only damages electrical equipment, but also contaminates the environment. Therefore, it is crucially important to effectively monitor and detect gas leakage for SF_6_ [4,5,6].

Traditional methods for detecting SF_6_ gas mainly include the soap bubble method, bandaging method, and electrochemical method [7,8,9,10]. However, these methods have low detection accuracy and real-time performance. Subsequently, infrared imaging technology has emerged as a research hotspot for detecting SF_6_ gas, offering high-accuracy, excellent real-time performance, and wide-ranging detection capabilities [11,12]. The most representative product is the GF306 gas imaging instrument developed by the American FLIR company (Manufacturer: FLIR Systems, Inc. City: Wilsonville, State abbreviation: OR, Country: USA) [13]. Meanwhile, many researchers have developed multispectral infrared imaging systems based on filtering wheels, which monitor various harmful gases in the atmosphere [14,15,16]. However, most existing infrared detection systems are equipped with only one filter to detect SF_6_ gas. The images captured contain background noise and system noise, making these systems vulnerable to interference from such noises [17,18,19], and the real-time performance of detection is limited by the rotation speed of the filtering wheel. Furthermore, gas detection methods such as frame differential [20], optical flow motion [21], and the mixed Gaussian background model [22,23] are utilized for monitoring the gas leakage. Nevertheless, the raw data from existing methods is in the video format, resulting in a large amount of data and complex computations.

Here, a method for monitoring SF_6_ gas leakage based on a customized binocular imaging (CBI) system is proposed. The CBI prototype is completed via assembling two customized filters into two cameras. The selection of the first filter is based on the absorption peak of SF_6_ gas, while the second filter is used to record background noise and system noise. Specially, the central wavelength of this second filter should keep away from the absorption resonant peaks of other gases, in order to avoid interference by them. Then, we calibrated the prototype to the ice, aiming to reduce the system noise. Subsequently, the difference method is used according to two infrared images captured by the CBI system to monitor the SF_6_ gas leakage. Finally, the detection effectiveness of our developed system is experimentally verified. Our customized prototype allows for its installation on unmanned platforms, thereby further extending the application scenes of the system.

## 2. Principle and Design

### 2.1. Conceptual Design of the CBI System

**Figure 1:**
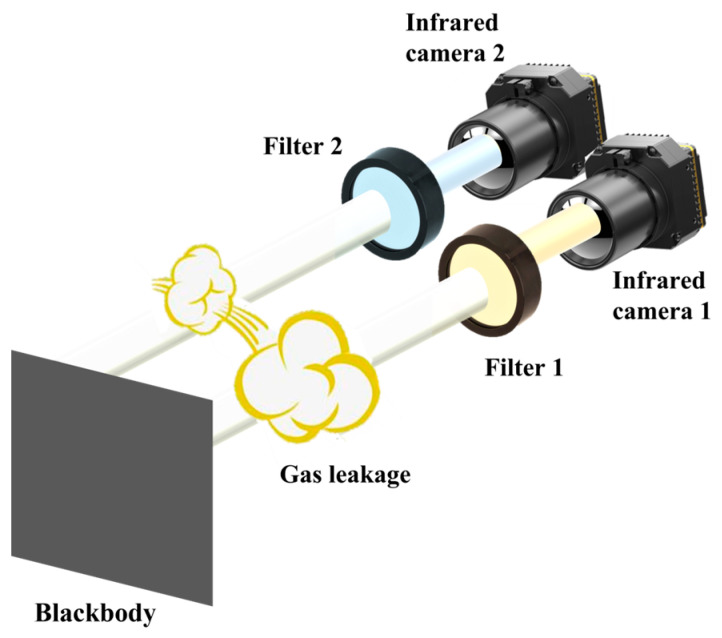
Schematic diagram of the CBI system.

A schematic diagram of the CBI system is shown in Figure 1. Based on the selective absorption characteristics of SF_6_ gas within the infrared band ranging from 8000 nm to 12,000 nm, which can be achieved by a blackbody radiation source as presented in our research, the obvious existence and distribution of SF_6_ gas can then be observed by employing the difference method to two infrared images, one considering its absorption resonant peak and the other considering its background interference. To fulfill this procedure, two infrared cameras of the same type were utilized, correspondingly configured with two customized optical filters in front of their objective lenses.

### 2.2. SF_6_ Gas Detection

**Figure 2:**
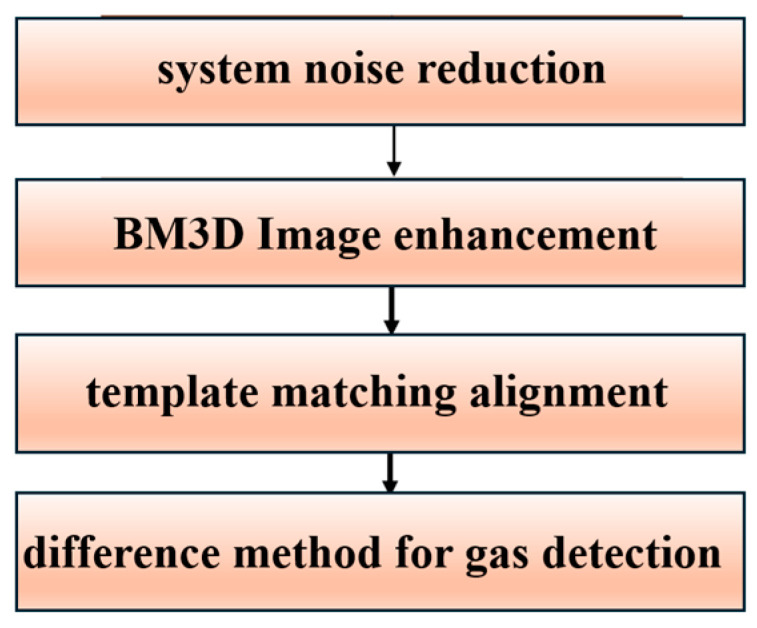
Flow chart of the image processing.

The major steps for detecting SF_6_ gas are demonstrated in Figure 2. Firstly, the CBI system is susceptible to system noise interference. The corrected output *DN*_out_ can be calculated using:

(1) $$DN_{\mathrm{out}}=DN_{\mathrm{target}}-DN_{\mathrm{dark}}$$

where *DN*_target_ and *DN*_dark_ represent the pixel values of target and dark current, respectively.

Secondly, the Block-Matching 3D (BM3D) algorithm is utilized for improving the image quality. This algorithm uses similar blocks in the image to reduce the noise, and employs multi-level processing to further enhance the denoising effect [24]. It can be mainly divided into grouping, collaborative filtering, and aggregation steps. During the grouping phase, reference blocks are selected from images, and other image blocks similar to the reference block are identified. During the collaborative filtering phase, these image blocks are filtered with a threshold to reduce the noise, while preserving important image details. During the aggregation phase, the processed image blocks are combined with the original image to obtain a new clear image.

Thirdly, since the CBI system has a binocular structure with a field-of-view difference between two cameras, template matching is employed by Equation (2). A small area with gas is selected from the first image as the template, and then a similar region is found when comparing all small areas in the second image with the template. The value of the correlation coefficient *ρ*(*x*, *y*) ranges from −1 to 1, with a value closer to one indicating a better similarity between the two areas.

(2) $$\rho (x,y)=\frac{\sigma (S_{x,y},g)}{\sqrt{D_{x,y}D}}$$

where *g* represents a template in the first image; *S_x_*_,*y*_ represents a sliding area in the second image, which has the same size as the template; $\sigma (S_{x,y,}\;g)$ is the covariance between *S_x_*_,*y*_ and *g*; and *D_x_*_,*y*_ and *D* are the variances of *S_x_*_,*y*_ and *g*, respectively.

Finally, the principle of the difference method can be expressed as follows:

(3) $$D\left(x,y\right)=\left\{ \begin{array}{l} S_{1}(x,y)-S_{2}(x,y),\;S_{1}(x,y)-S_{2}(x,y)>w\\ 0,\;S_{1}(x,y)-S_{2}(x,y)\leq w \end{array}\right.$$

where *S*_1_(*x*,*y*) and *S*_2_(*x*,*y*) represent the first and the second images, and *D*(*x*,*y*) denotes the differential image. It is worth noting that the obvious existence and the distribution of SF_6_ gas are detected by employing the difference method to two infrared images.

The adaptive threshold *w* is determined by the trial-and-error method, considering different blackbody temperatures. Each pixel value in the differential image is compared with the threshold value. When the pixel value is larger, it is identified as the target, otherwise it is identified as the background.

## 3. Results and Discussion

### 3.1. Development of the CBI System

**Figure 3:**
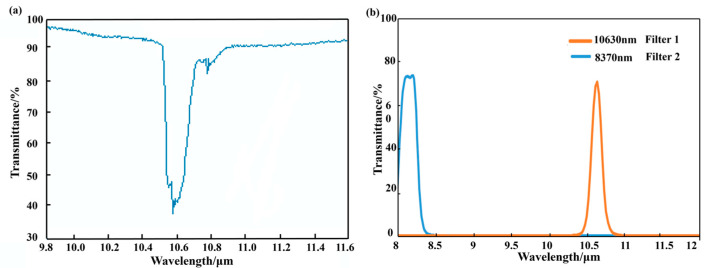
(**a**) Spectral transmittance curve of SF_6_ gas. (**b**) Spectral transmittance curves of two customized filters.

The central wavelength of the first filter corresponds to the absorption resonant peak of SF_6_ gas. However, the image captured also contains background noise and system noise. As a result, the second filter is used to record all these noises. One aspect to note is that, in order to avoid the interference by other gases, the central wavelength of this second filter should keep away from the absorption resonant peaks of all those gases, especially those in the covered infrared band from 8000 nm to 12,000 nm. We installed multiple bandpass filters in front of the infrared cameras for a contrast comparison, ensuring that these filters conform to the above selection condition for the second filter. Ultimately, 8370 nm was determined as the central wavelength. Meanwhile, analyzing the data from the HITRAN database [25], the infrared spectral transmittance of SF_6_ gas is shown in Figure 3a, which reveals a prominent absorption peak at the wavelength of 10,630 nm. Hence, we selected 10,630 nm as the central wavelength for the first filter. The spectral transmittance curves of the two customized filters are depicted in Figure 3b.

**Figure 4:**
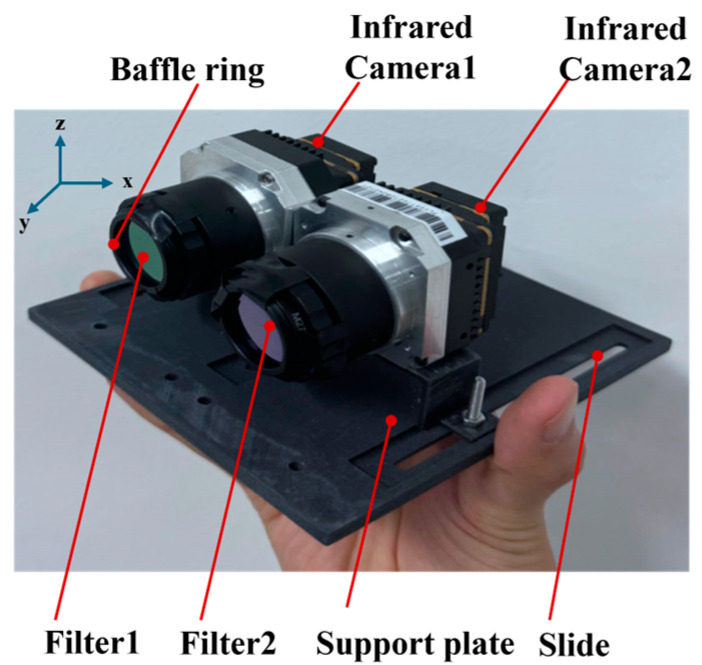
The composition of the CBI prototype.

The composition of the CBI prototype with dimensions of 120 mm × 130 mm × 90 mm is illustrated in Figure 4. Two customized filters are separately assembled with two cameras of the same type, installed adjacent to each other, thus accomplishing the CBI prototype. The parameters of the customized system are illustrated in Table 1. Our developed system supports a plug-and-play functionality. The first filter of the CBI system, which determines the type of gas, can be readily replaced to accommodate various gas detection tasks.

### 3.2. SF_6_ Gas Detection Experiments

To demonstrate the SF_6_ gas detection capability of our developed prototype, different blackbody temperatures (30 °C, 40 °C, 50 °C, and 60 °C) served as the illumination light source. The distance between our system and the gas was 2 m. The exposure time of each camera was set to the same. SF_6_ gas was released into the air from a container with an adjustable pressure.

**Figure 5:**
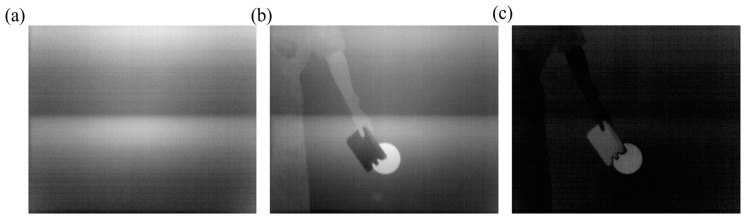
System noise reduction results. (**a**) The image of the dark current. (**b**) The uncorrected image. (**c**) The corrected image.

Firstly, the system noise was reduced based on Equation (1). We approximated the temperature of the ice to 0 °C. Then, we placed a piece of ice close to the camera and obtained the pixel values for the dark current *DN*_dark_. Subsequently, we removed the ice and acquired the pixel values for the target *DN*_target_. The images of the dark current and the target are shown in Figure 5a,b. The corrected image of the target is displayed in Figure 5c, significantly decreasing the system noise.

**Figure 6:**
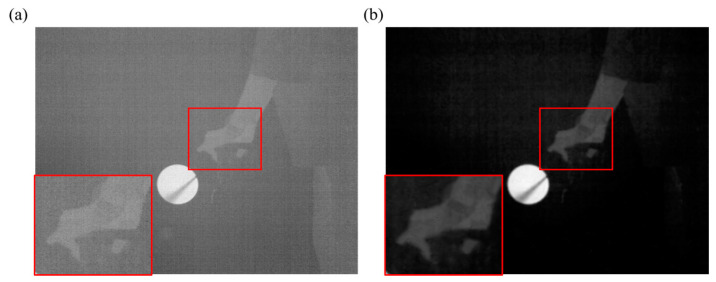
(**a**) The raw image. (**b**) The enhanced image using the BM3D algorithm. (The texture information of the hand within the red box is more prominent).

**Figure 7:**
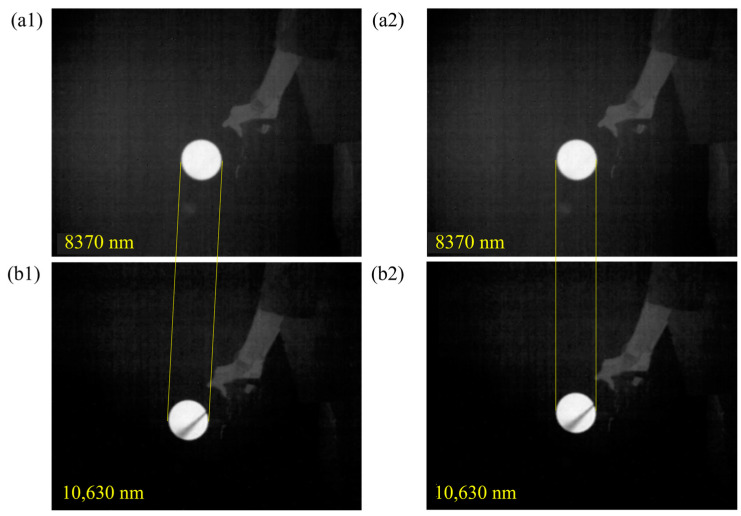
The images (**a**) before and (**b**) after the template matching alignment.

Secondly, we utilized the BM3D algorithm to improve the image quality. The results are depicted in Figure 6. Compared to the raw image, vertical stripes are significantly reduced, and the background is more uniform in the enhanced image. Next, template matching is used to compensate for the field-of-view differences, as shown in Figure 7. This procedure can accurately align the SF_6_ gas regions in these two images.

**Figure 8:**
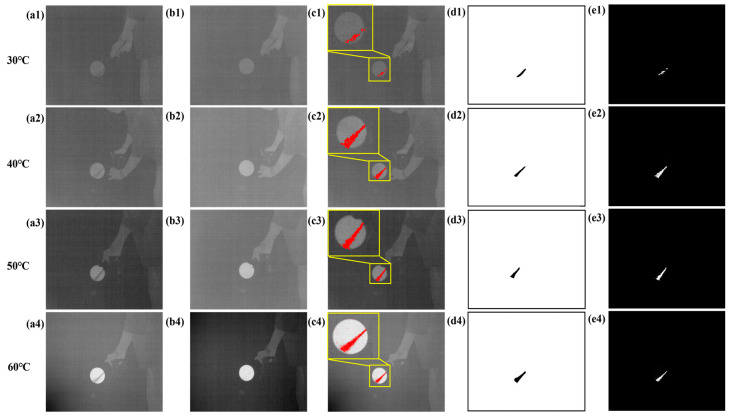
The detection results of SF_6_ gas in different background temperatures using the CBI system. (**a1**–**a4**) The images by the first filter (10,630 nm) in different background temperatures. (**b1**–**b4**) The images by the second filter (8370 nm) in different background temperatures. (**c1**–**c4**) The detection results in different background temperatures. (**d1**–**d4**) The ground-truth labels of SF_6_ gas in different background temperatures. (**e1**–**e4**) The measured labels of SF_6_ gas using our developed method in different background temperatures.

Finally, we utilized the difference method to detect the leaking plume of SF_6_ gas. As SF_6_ gas has a molecular weight larger than that of air, it moves to the ground. The images obtained by our developed prototype in Figure 8a,b show significant pixel value differences in the regions containing the gas. In Figure 8c, SF_6_ gas can be easily observed using the difference method. The position revealing the gas leakage is marked in red. Three sets of experiments for verifying the repeatability were performed under identical temperature conditions. To quantify the effectiveness of the gas detection, the ground-truth labels and the measured labels of SF_6_ gas are shown in Figure 8d,e. Then, the accuracy can be evaluated using Equation (4), as depicted in Table 2. Finally, the average accuracy of the three sets of experiments is obtained (see Table 2).

(4) $$Accuracy=\frac{\mathrm{TP}+\mathrm{TN}}{N}$$

where, TP and TN are the number of pixels of true target and background detections, according to the comparison of the pixels between the ground-truth labels (Figure 8d) and the measured labels (Figure 8e). N is the total number of pixels.

Temperatures of 30 °C, 40 °C, 50 °C, and 60 °C represent the blackbody temperatures serving as the illumination light source. Higher blackbody temperatures result in stronger infrared radiation, consequently enhancing the signal-to-noise ratio (SNR) of SF_6_ gas in the image. This increased SNR improves the visibility of the gas in the captured infrared image. As illustrated in Figure 8 and Table 2, the accuracy remains consistently above 99.8%, showing slight variations with increased radiation energy. Therefore, our customized prototype can effectively detect SF_6_ gas leakage.

## 4. Conclusions

In summary, we propose a method for monitoring the SF_6_ gas leakage based on a customized binocular imaging system. Firstly, we determine the central wavelength of the first filter as 10,630 nm based on the absorption resonant peak of SF_6_ gas. The second filter is used to record background noise and system noise. Specifically, the central wavelength of this second filter is determined as 8370 nm, keeping away from the absorption resonant peaks of all other gases, avoiding the interference by these gases. Secondly, two customized filters are discretely assembled with two cameras, and the CBI system is accomplished. Thirdly, we reduced system noise by calibrating the prototype to ice and using the BM3D algorithm. Then, since the CBI system has a binocular structure with a field-of-view difference between the two cameras, template matching is employed to determine the alignment. Finally, we utilize the difference method to detect the gas. The results show that our developed system detects SF_6_ gas with a high accuracy of over 99.8%. Compared to existing SF_6_ gas detection methods, our customized system has two channels, significantly reducing the interference of system noise and background noise. Furthermore, the CBI system supports a plug-and-play customization for detecting multiple gases, to increase its flexibility and universality. The proposed method can also be simply configured via employing a filtering wheel and a cooled focal plane array (FPA) detector for remote sensing purposes.

## Figures and Tables

**Table 1:** Parameter overview of the CBI system.

Parameters	Value
Spectral range	8000–14,000 nm
Channels	2 (10,630 nm, 8370 nm)
NETD	<30 mk
Image resolution	640 × 512 pixels
Field of view	80°
Focal length	4.2 mm
Dimensions	120 mm × 130 mm × 90 mm

**Table 2:** Quantitative indictors of the CBI system in four different temperatures of the blackbody.

Temperature	Accuracy
30 °C	99.88%
40 °C	99.88%
50 °C	99.92%
60 °C	99.83%

## Data Availability

All data underlying this article are available in the article.
